# Impact of Serum Albumin Levels on Prognosis and Recurrence in Patients with Hepatocellular Carcinoma

**DOI:** 10.3390/cancers17182971

**Published:** 2025-09-11

**Authors:** Naoko Hayata, Atsushi Hosui, Tomohide Kurahashi, Shigeki Suemura, Akane Namiki, Akino Okamoto, Takafumi Tanimoto, Hiroki Murai, Kohsaku Ohnishi, Motohiro Hirao, Takuya Yamada, Naoki Hiramatsu

**Affiliations:** Department of Gastroenterology and Hepatology, Osaka Rosai Hospital, 1179-3 Nagasonecho, Kita Ward, Sakai 591-8025, Osaka, Japan

**Keywords:** albumin, liver function, tumor invasion, serosal invasion, HCC, prognosis, progression-free survival

## Abstract

**Background**: Liver function is a critical factor, both in the selection of treatment and in the prediction of prognosis in patients with hepatocellular carcinoma (HCC). The ALBI grade, introduced as a more objective method of assessing liver function, utilizes serum albumin (Alb) and total bilirubin (Bil) levels. Although albumin is widely recognized for its role in maintaining colloid osmotic pressure and regulating plasma volume, recent studies have implicated it in tumor progression, invasion, and metastasis. The purpose of this study is to determine the impact of serum albumin levels on overall survival (OS) and tumor invasion/metastasis in HCC patients with the same liver function (ALBI grade) at the time of diagnosis. **Methods**: In this study, 285 patients diagnosed with primary HCC at our institution from 2015 to 2019 were classified by ALBI grade and analyzed. Among them, 78 patients with ALBI grade 2 status were selected to evaluate the impact of albumin level. To further isolate the effect of albumin rather than bilirubin, patients in the ALBI grade 2 cohort were divided into two groups based on mean values of Alb (3.5 g/dL) and Bil (1.0 mg/dL). Alb normal group (Group A): Alb ≥ 3.5 g/dL, Bil ≥ 1.0 mg/dL (n = 42). Bil normal group (Group T): Alb < 3.5 g/dL, Bil < 1.0 mg/dL (n = 36). Liver function was almost the same in these two groups based on the ALBI grade. OS, progression-free survival (PFS), types of recurrence, and pathological findings were compared between the two groups. OS was analyzed by the log-rank test, and comparisons between the two groups were performed by the *t*-test and chi-square test, with *p* < 0.05 indicating statistical significance. **Results**: OS was significantly worse in Group T than in Group A before and after propensity score matching based on age, performance status, and HCC stage (*p* < 0.001 and *p* = 0.011). Among the 44 patients who received curative treatment (surgery or radiofrequency ablation), OS was also significantly worse in Group T (*p* < 0.001). An analysis of the recurrence patterns of 44 curatively treated patients revealed that Group T had significantly shorter PFS (*p* < 0.001), and all recurrence patterns were multiple (*p* = 0.002). Pathological analysis in 28 surgical patients showed that serosal invasion was present in significantly more patients in Group T (*p* = 0.003). **Conclusions**: Low serum albumin levels in patients with HCC indicate both liver dysfunction and increased tumor invasion and metastasis. Nutritional support and albumin supplementation may help reduce intrahepatic metastases and improve prognosis. Further studies are needed to explore the underlying mechanisms and therapeutic potential.

## Simple Summary

Liver function is a critical factor, both in the selection of treatment and in the prediction of prognosis in patients with hepatocellular carcinoma. The ALBI grade, introduced as a more objective method of assessing liver function, utilizes serum albumin and total bilirubin levels. Although albumin is widely recognized for its role in maintaining colloid osmotic pressure and regulating plasma volume, recent studies have implicated it in tumor progression, invasion, and metastasis. Among 285 patients diagnosed with primary HCC, 78 patients with ALBI grade 2 were selected and further divided into two groups based on mean values of Alb and Bil: Liver function was almost same in these two groups based on the ALBI grade. OS, progression-free survival, types of recurrence, and pathological findings were compared between the two groups. Low albumin levels may not only indicate deteriorating liver function but also increase the risk of cancer invasion and metastasis. In HCC patients with Alb < 3.5 g/dL, nutritional improvement and albumin supplementation may reduce intrahepatic metastasis and invasion, ultimately improving prognosis.

## 1. Introduction

In patients with hepatocellular carcinoma (HCC), liver function is a crucial factor, both in the choice of treatment and in prediction of prognosis. The Child–Pugh classification has been widely used in the assessment of liver function, but it has several problems [1,2]. First, it contains overlapping factors; that is, ascites are accompanied by albumin (Alb) levels. Second, ascites and encephalopathy are subjective factors and difficult to quantify. Therefore, Johnson PJ et al. proposed the albumin-bilirubin (ALBI) grade as a new method to evaluate liver function in 2015. This is calculated using only objective blood tests assessing Alb and bilirubin (Bil) levels [3]. Yamana et al. reported that the 1-year and 5-year cumulative survival rates of patients with ALBI grades 1, 2, and 3 were 98% and 80%, 91% and 56%, and 58% and 23%, respectively [4]. Kudo M et al. showed that overall survival was longer in HCC patients with better ALBI grades [5]. However, the ALBI grade has a significant limitation in that Grade 2 encompasses a wide range of patients, including those with mild to moderate hepatic impairment, resulting in considerable heterogeneity in prognosis. To address this limitation, Hirayama et al. proposed the modified ALBI (mALBI) grade, which subdivides Grade 2 into 2a and 2b, allowing for more accurate prognostic stratification [6].

Albumin is a protein synthesized in the liver that accounts for approximately 60% of plasma proteins, and is more abundant than any of the other 100 known plasma proteins [7,8,9]. The main function of albumin is to maintain colloid osmolarity and to regulate plasma volume [10]. In addition, albumin reversibly binds to many molecules, including drugs, metal ions, and multiple inflammatory mediators, and can affect systemic inflammation, immune response, antioxidant capacity, and endothelial cells [11,12,13,14,15]. Therefore, not only does albumin play a role as an indicator of liver function, but the albumin protein itself is known to improve the prognosis of cirrhosis in patients [16]. There are also reports that albumin inhibits cancer invasion and metastasis, indicating its involvement in the progression of HCC [17,18].

Disentangling the prognostic implications of hypoalbuminemia—whether stemming from poor liver function or a direct influence on tumor biology—remains a major challenge. Few studies clearly differentiate these intertwined effects, leaving open the question of whether albumin hinders HCC progression or merely serves as a surrogate for hepatic insufficiency. This gap underscores the need for focused mechanistic and clinical research to clarify albumin’s dual roles in HCC.

To determine whether the albumin concentration improves the prognosis of HCC in patients, we aimed to determine the effect of Alb concentration on overall survival (OS), tumor invasion and metastasis in patients with the same liver function (ALBI grade) at the time of primary HCC diagnosis.

Treatment strategies were as follows:

Treatment strategies for HCC are guided by the Barcelona Clinic Liver Cancer (BCLC) staging system, which incorporates tumor size and number, liver function, and patient performance status (PS). The BCLC system provides a framework for stage-specific treatment recommendations that are endorsed by major clinical guidelines, including those from the American Association for the Study of Liver Diseases (AASLD) and the Japan Society of Hepatology (JSH). For early-stage HCC (BCLC stage 0 or A), curative treatments such as surgical resection or radiofrequency ablation (RFA), are recommended. In intermediate-stage disease (BCLC B), transarterial chemoembolization (TACE) is considered the standard care. These guidelines aim to standardize clinical decision-making while accounting for regional differences in patient populations and available resources. Treatment selection often requires a multidisciplinary approach tailored to individual patient characteristics and institutional practices.

TACE therapy: The femoral artery was punctured via the Seldinger technique, and a 5-F catheter and/or a microcatheter were placed in the tumor supply vessels. Then, 5–10 mL of lipiodol and 20–60 mg of epirubicin were mixed into an emulsion and slowly injected.

RFA therapy: RFA was performed via an Arfa (Japan Lifeline Co., Ltd., Tokyo, Japan) RFA system with an adjustable electrode needle. All RFA sessions were performed via a percutaneous approach under ultrasound guidance (LOGIQ E9 XDclear 2.0, GE Healthcare, Chicago, IL, USA).

Operational Definitions: HCC stage classification: Accurate staging of HCC is critical for assessing prognosis and determining appropriate treatment strategies. Among various staging systems, the Tumor-Node-Metastasis (TNM) classification, established by the American Joint Committee on Cancer (AJCC) and the Union for International Cancer Control (UICC), is one of the most widely used frameworks for cancer staging, including HCC. The TNM system categorizes the extent of the disease based on three components: the size and number of primary tumors (T), regional lymph node involvement (N), and the presence of distant metastases (M). In HCC, the T category also incorporates features such as vascular invasion, which is a strong predictor of recurrence and poor survival. Stages 1–4 were finally determined based on the overall judgment regarding T, N, and M classifications.

The 8th edition of the AJCC TNM classification refined earlier versions by distinguishing between solitary tumors with and without vascular invasion, and by defining multiple tumors and macrovascular invasion more precisely. Although the TNM system focuses on tumor burden rather than liver function, it remains a valuable tool, especially in surgical and oncological settings where histopathological evaluation is available. However, its prognostic utility may be limited in patients with advanced cirrhosis, where liver function, often assessed by Child–Pugh or ALBI scores, also significantly impacts outcomes.

## 2. Materials and Methods

From January 2015 to December 2019, 285 cases were diagnosed with primary HCC at this institution. The inclusion criteria for this retrospective study were as follows: (1) HCC diagnosed on the basis of computed tomography (CT) and magnetic resonance imaging (MRI) or histologically proven in a biopsy specimen; (2) observatory periods for more than six months; and (3) availability of clinical data, including the therapeutic response. The exclusion criteria were as follows: (4) disease complicated with other malignant tumors; (5) history of organ transplantation; and (6) serious complications, including severe heart (e.g., myocardial infarction or heart failure), lung, kidney (e.g., nephrotic syndrome or severe proteinuria), or blood coagulation dysfunction.

**Figure 1:**
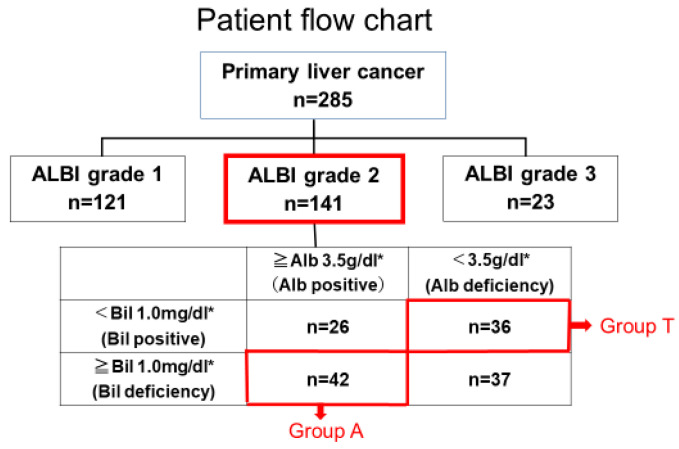
Patient flow chart. * is the mean value in patients with ALBI grade 2.

Among these 285 patients with primary HCC, 121 cases were classified as ALBI grade 1, 141 as ALBI grade 2, and 23 as ALBI grade 3. The focus of this study was 141 cases with ALBI grade 2 status and the impact of serum albumin levels while maintaining the same liver function. To further isolate the influence of Alb rather than Bil, we classified patients into four groups based on the mean values of Alb (3.5 g/dL) and Bil (1.0 mg/dL) in the ALBI grade 2 cohort (Figure 1):

Among these, patients with high albumin and low bilirubin, as well as those with low albumin and high bilirubin, were considered inappropriate for comparison under the assumption of equal liver function. Furthermore, it was deemed difficult to determine which factor—albumin or bilirubin—primarily influenced prognosis in these subgroups. Therefore, these two groups were excluded from the analysis. We limited our evaluation to two groups with more homogeneous liver function, in which the individual effects of albumin or bilirubin could be more clearly assessed.

Patients with albumin ≥ 3.5 g/dL and bilirubin ≥ 1.0 mg/dL were defined as Group A, while those with albumin < 3.5 g/dL and bilirubin < 1.0 mg/dL were defined as Group T. These two groups were compared in our analysis:

Group A (albumin-preserved group): Alb ≥ 3.5 g/dL and Bil ≥ 1.0 mg/dL (n = 42)
Group T (bilirubin-preserved group): Alb < 3.5 g/dL and Bil < 1.0 mg/dL (n = 36)

Overall survival (OS), tumor stage, maximum tumor diameter, number of tumors, initial treatment modality, and postoperative pathological findings were compared between Group A and Group T.

Statistical analysis. Survival analysis was performed using the log-rank test, whereas the *t*-test and χ^2^ test were applied for comparisons between the two groups. A difference in the data with a *p*-value < 0.05 was considered to indicate statistical significance. All the statistical analyses were performed with JMP software (version 12, SAS Institute Japan, Tokyo, Japan).

Ethics statement. This study was approved by the Review Board of Osaka Rosai Hospital (2023-201) and conducted in accordance with the principles of the Declaration of Helsinki. Written informed consent was obtained from all the patients.

## 3. Results

### 3.1. Clinical Background in Groups a and T with the Same Liver Function

**Figure 2:**
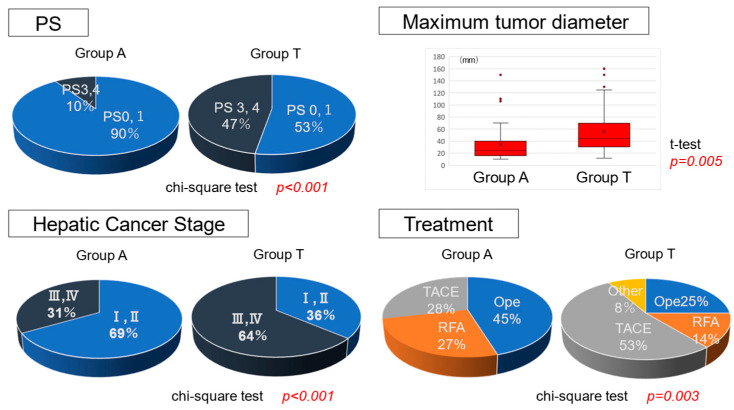
Comparison of tumor factors between the two groups.

**Figure 3:**
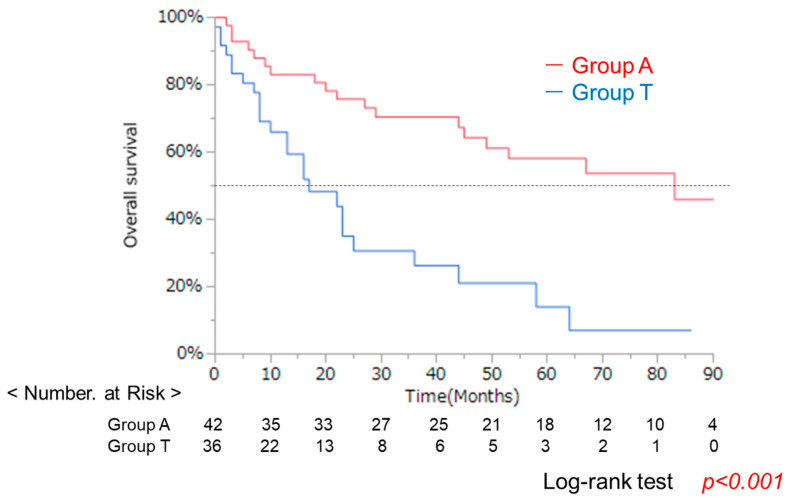
Overall survival (OS) of 78 patients. Dash line shows 50%.

The clinical characteristics of the 78 patients with same liver function classified as ALBI grade 2 are summarized in Table 1. The median age was significantly younger in Group A (70.5, 55–88) than in Group T (75.6, 63–98). No significant differences were observed in sex, etiology (viral/non-viral), platelet count, or PT values related to liver function. A comparison of performance status (PS) revealed a significantly higher proportion of patients with PS ≥ 2 in Group T. More advanced cases (stage III/IV) were also observed in Group T (Figure 2). Additionally, the maximum tumor diameter was significantly larger in Group T. Fewer patients received curative treatments, such as surgery or radiofrequency ablation (RFA), whereas non-curative treatments, such as TACE, were significantly more common in Group T. OS was significantly worse in Group T than in Group A (*p* < 0.001) (Figure 3).

A difference in age was observed between the two groups. Additionally, in Group T, performance status (PS) was significantly poor, and there were significantly more patients with advanced liver cancer stages. Patients with larger maximum tumor diameters were significantly more common in Group T, and the number of patients who underwent curative treatments (surgery or RFA) was significantly lower. Kaplan–Meier analysis and the log-rank test demonstrated that OS was significantly worse in Group T than in Group A. An imbalance in clinical backgrounds between the two groups led to the use of propensity score matching to minimize confounding.

Analysis of overall survival (OS) using the Kaplan–Meier method demonstrated that Group T had a significantly lower survival rate compared to Group A (log-rank test, *p* < 0.001). However, clinical backgrounds differed substantially between groups, and this unadjusted analysis may be influenced by confounding factors.

### 3.2. Clinical Background and OS After Propensity Score Matching

**Figure 4:**
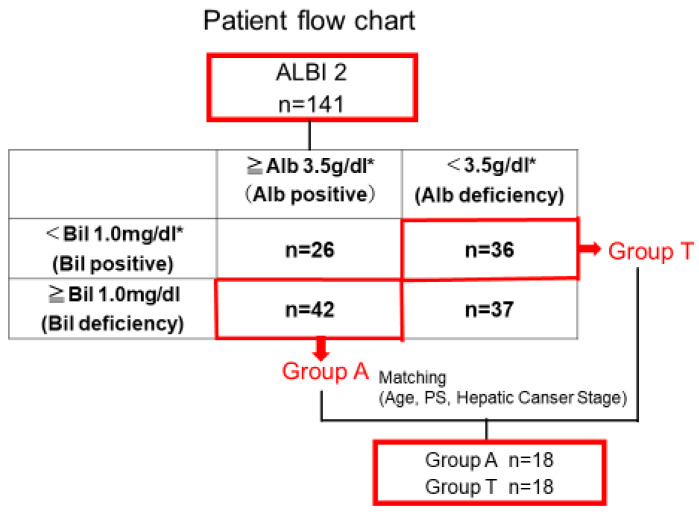
Patient flow chart after propensity score matching. * is the mean value in patients with ALBI grade 2.

**Figure 5:**
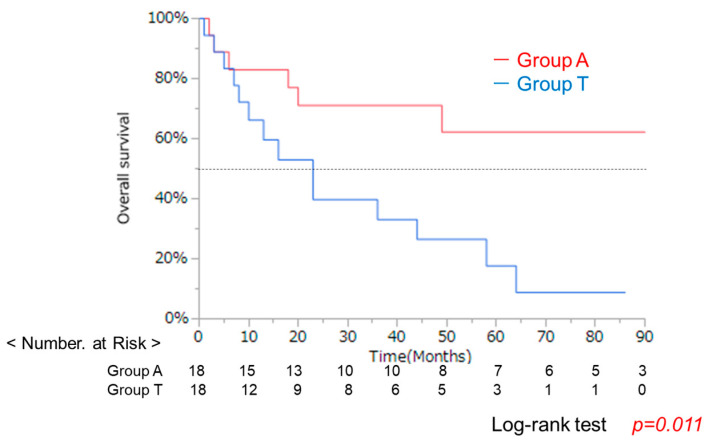
OS of 36 patients after PSM.

Group T had disadvantageous backgrounds, such as older age, advanced stage of HCC, poor PS. To match patients according to their clinical background, age, PS, and clinical stage of liver cancer, propensity score matching (PSM) was performed, yielding 18 patients each in Group A and Group T (Figure 4). In this matched cohort, PT values were significantly higher in Group T (Table 2), and OS remained significantly worse in Group T (*p* = 0.011) (Figure 5). These results indicate that low albumin itself is related to larger tumor and poor prognosis even if PT values were significantly better in low-albumin group.

A total of 42 patients from Group A and 36 patients from Group T after propensity score matching based on age, PS, and liver cancer stage. After matching, 18 patients were selected from each group.

Analysis of overall survival (OS) using the Kaplan–Meier method demonstrated that Group T had a significantly lower survival rate compared to Group A (log-rank test, *p* = 0.011).

### 3.3. Clincal Background and OS in Groups a and T After Curative Treatment

**Figure 6:**
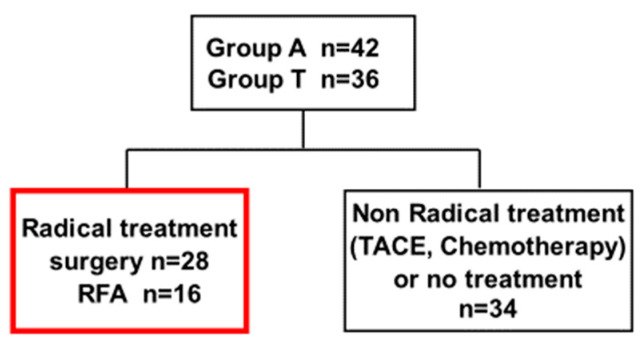
Patient flow chart of 44 patients who underwent curative treatment (surgery or RFA).

**Figure 7:**
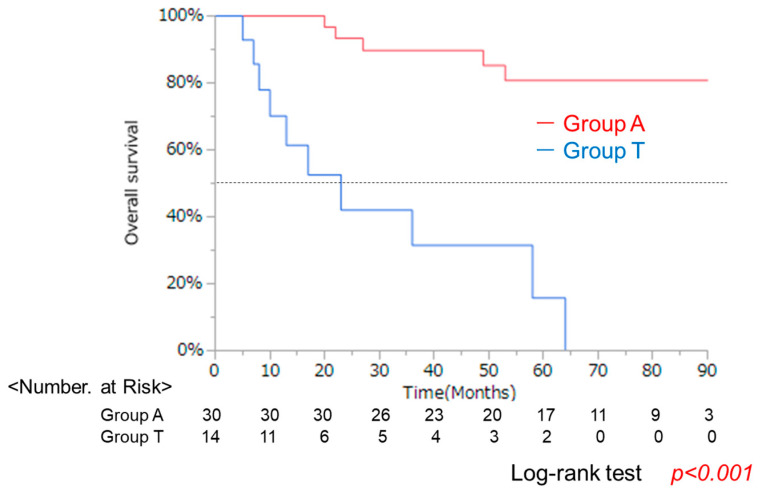
OS of 44 patients under curative treatment.

Poor prognosis may be due to the more advanced cases and larger tumors in Group T. Next, we focused on patients with curative treatment. A subgroup analysis was conducted on 44 patients that received curative treatments (surgery or RFA), comprising 28 surgical and 16 RFA cases (Figure 6). The maximum tumor diameter tended to be larger in Group T, but no significant differences were found in age, sex, HCC stage, or liver function except for the Bil and Alb values (Table 3). OS was significantly worse in Group T (*p* < 0.0001) (Figure 7). These results suggested that lower albumin was associated with poor prognosis even after curative therapy. At the same time, the reason for poor prognosis cannot be explained simply by the progression of liver tumors in lower albumin group.

Analysis of overall survival (OS) using the Kaplan–Meier method demonstrated that Group T had a significantly lower survival rate compared to Group A (log-rank test, *p* < 0.001).

### 3.4. Clinical Background and OS in Groups a and T After RFA

**Figure 8:**
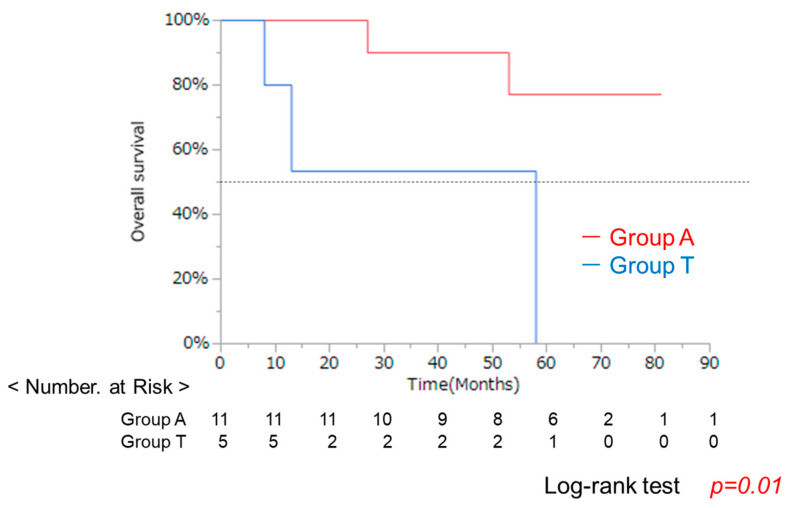
OS of 16 patients who underwent RFA.

Indications for RFA are determined by guidelines from various societies, and the backgrounds of eligible patients are likely to be similar. Further analysis of the 16 RFA cases revealed a significant difference in sex (*p* = 0.007), but no other significant differences as expected (Table 4). OS remained significantly worse in Group T (*p* = 0.01) (Figure 8). These results also suggest that lower albumin was associated with poor prognosis even after RFA therapy. As described in the above session, the reason for poor prognosis cannot be explained simply by the progression of liver tumors in lower albumin group.

Analysis of overall survival (OS) using the Kaplan–Meier method demonstrated that Group T had a significantly lower survival rate compared to Group A (log-rank test, *p* = 0.01).

### 3.5. Comparison of Recurrence-Free Survival and Recurrence Patterns in 44 Patients Receiving Curative Treatment (Surgical Resection or Radiofrequency Ablation)

**Figure 9:**
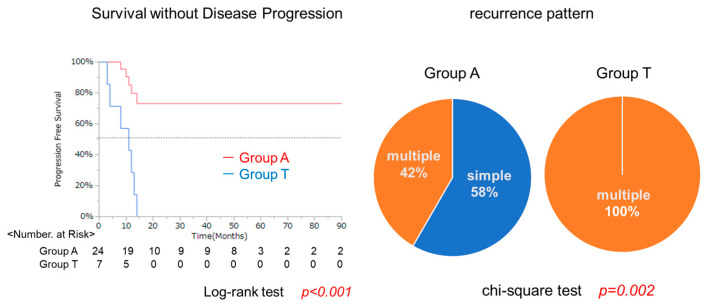
Comparison of recurrence-free survival and recurrence patterns in 44 patients receiving curative treatment (surgical resection or radiofrequency ablation).

Next, we focused on the clinical course of these patients after curative treatment. Recurrence after initial treatment for HCC was analyzed, and thirty-one patients had recurrence. The time to recurrence was significantly shorter in Group T than in Group A (Figure 9, left panel). With respect to recurrence patterns, solitary recurrence was more frequent in the normal Alb group, whereas all cases in Group T developed multiple recurrences (Figure 9, right panel). These results indicate that lower albumin had influences on the development or invasion of HCC. This is a critical point of this study. The clinical course becomes different according to albumin level and it might be possible to make these patients have better prognosis when albumin levels were normalized.

Kaplan–Meier analysis of overall survival (OS) showed that Group T had a significantly lower survival rate compared to Group A (log-rank test, *p* < 0.001). Additionally, while 42% of patients in Group A experienced multiple recurrences, all patients in Group T developed multiple recurrences.5.6. Histological Study in Surgical Specimens in Group A and T

### 3.6. Histological Study in Surgical Specimens in Group A and T

**Figure 10:**
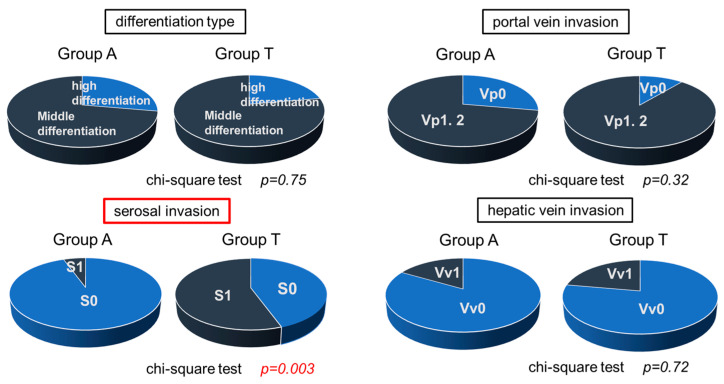
Comparison of surgical samples from 28 patients who underwent surgery.

To clarify the reason why multiple recurrences were found in a shorter time after curative treatment, surgical samples from 28 resected tissues were further examined, including 19 from Group A and 9 from Group T. In the analysis of the 28 surgical cases, no significant differences were observed in patient background between Group A and Group T (Table 5). Overall, portal vein invasion was observed in 78% of the total samples. No significant differences were found in differentiation type (well/moderately differentiated), portal vein invasion, or hepatic vein invasion. However, serosal invasion was significantly more common in Group T (*p* = 0.003) (Figure 10). These results suggested that serosal invasion might cause multiple recurrences. Significant difference was only found in frequency of serosal invasion, but portal invasion and hepatic invasion were also higher in lower albumin group. Infiltration of carcinoma cells might be easier in the state of lower albumin, and it may cause poor prognosis.

No significant differences in tumor differentiation (well-differentiated/moderately differentiated), the presence or absence of portal vein invasion, or hepatic vein invasion were detected between the two groups. However, serosal invasion was significantly more common in Group T.

## 4. Discussion

In cirrhotic patients, hypoalbuminemia results from decreased albumin synthesis and catabolic enhancement due to structural changes in the albumin molecule. Basically, albumin exhibits antioxidant properties, and its administration has been shown to mitigate oxidative stress. In the circulation, albumin predominantly exists in the reduced form, which plays a crucial role in scavenging reactive oxygen species and reactive nitrogen species [19]. These structural and functional abnormalities, including change from reduced form to oxidized form, were caused by post-transcriptional modifications and oxidative changes, leading to inflammation and oxidative stress, thereby adversely affecting DNA integrity, cell proliferation, and apoptosis, and are considered to contribute to development of HCC [20]. Indeed, mutations in cancer-related genes such as TERT, TP53, and CTNNB1 have been reported to be induced by oxidative stress. Moreover, the release of inflammatory cytokines and chemokines promotes the formation of the tumor microenvironment, further facilitating HCC development and invasion [21].

The mALBI grade is already widely used to further stratify ALBI grade 2 patients and improve prognostic accuracy. However, mALBI was not utilized for the selection of this study population. The aim of this study was to clarify the biological role of albumin, so enough patients with almost same liver function were only needed. Thus, we decided to use ALBI grade not mALBI grade for our study. The novelty of this study lies in focusing on the biological role of albumin itself, rather than on further stratification of ALBI grade 2.

Regarding the correction of the differences in clinical background, we would like to elaborate on the rationale for including age in the matching process. Age is a clinically relevant factor that can indirectly influence outcomes through its effect on treatment selection, patient tolerance, and the presence of comorbidities. Although its direct influence on tumor biology may be limited, failing to account for age could introduce confounding, particularly in retrospective analyses where treatment allocation is not randomized. By incorporating age as a matching variable, we aimed to mitigate this potential bias and enhance comparability between groups. We recognize, however, that our matching approach has limitations. Specifically, the staging spectrum in our study population was broad, and while matching reduced imbalances in certain baseline characteristics, residual differences may persist. Such imbalances could still influence both treatment decisions and clinical outcomes, potentially affecting the interpretation of our findings. Furthermore, matching on age cannot fully account for other unmeasured confounders related to disease aggressiveness or patient fitness.

Caraceni et al. conducted a randomized trial in 431 cirrhotic patients and demonstrated that infusion of albumin itself improved survival outcomes [16]. Treatment with albumin had other benefits on improvement of the status of diseases, for instance, spontaneous bacterial peritonitis [22,23]. Furthermore, Muto et al. reported that, in decompensated cirrhotic patients, the administration of branched-chain amino acids (BCAA) significantly increased albumin levels and reduced progression to hepatic failure compared with diet therapy alone [24]. In obese cirrhotic patients (BMI ≥ 25), BCAA administration also significantly reduces the incidence of HCC, suggesting its potential as an anti-cancer agent [25,26].

Several studies have shown that hypoalbuminemia is associated with tumor growth, metastasis, and poor prognosis in various cancers, including gastric, colorectal, lung, gynecological, and breast cancers, as well as in patients with hepatocellular carcinoma [27,28,29,30,31,32,33,34,35,36,37,38]. In our study, the Alb-low patients presented larger tumor sizes, more frequent multiple recurrences, and shorter OS than the Alb-normal patients did. Tumor invasion and metastasis mechanisms commonly involve urokinase-type plasminogen activator (uPA) and its receptor (uPAR), which activate matrix metalloproteinases (MMPs) and degrade the extracellular matrix, facilitating cancer cell invasion [17,39].

A lot of investigators explained that low albumin is related to cell cycle and diameters of tumors [27,40,41]. Xiao et al. demonstrated that Alb suppression in HCC cell lines increased the expression of MMP2, MMP9, and uPAR, which are involved in epithelial–mesenchymal transition (EMT), suggesting that Alb interacts with uPAR to inhibit HCC invasion and metastasis [17].

Sonohara et al. reported that in patients who underwent resection of HCC, recurrence was more frequent in cases with hypoalbuminemia and that serosal or vascular invasion led to worsened OS and PFS [42]. Several investigators also showed that serosal invasion is related to albumin concentration in other types of cancer [43,44].

According to Kagi, the hepatic lymphatic system consists of three pathways: superficial subserosal lymphatic vessels, deep interlobular connective tissue lymphatic vessels, and hepatic venous system lymphatic vessels. These pathways either descend toward the hepatic hilum or ascend toward the entrance of the inferior vena cava. Lymphatic vessels are abundant near hepatocellular carcinoma and liver metastases, and when tumor tissue reaches the serosa, metastasis via the lymphatic vessels is thought to occur more easily [45].

Furthermore, the subserosal lymphatic vessels, interlobular lymphatic vessels, and hepatic venous system lymphatic vessels are closely interconnected, suggesting that they may influence not only lymph node metastasis but also synchronous and metachronous liver metastases [46]. Reports on other cancers, such as gastric cancer, have also indicated a correlation between albumin levels and serosal invasion. These findings suggest that low albumin levels may increase the risk of serosal invasion and promote metastasis. However, further research is needed to clarify the relationship between albumin levels and serosal invasion, frequency of recurrence, and prognosis of patients with primary HCC.

Our results suggest that administration of albumin not only produces a temporary increase in albumin concentration but also works well against HCC. Therefore, it is not desirable to neglect hypoalbuminemia in patients with HCC for the purpose of improving prognosis.

This study has several limitations. First, the relatively small sample size may limit the generalizability of the findings. The possibility cannot be excluded that confounding factors may have influenced the results. Second, the retrospective study design and single-center setting may introduce selection bias. Third, factors causing hypoalbuminemia other than liver disease, such as malnutrition or inflammatory state, may not have been fully excluded because inflammation markers (C-reactive protein) or Controlling Nutrition Status scores were not compared [47]. Poor nutrition or an inflammatory state may have influenced our results. Fourth, this study included patients diagnosed with HCC from 2015 to 2019, our results may weaken the clinical relevance of the study because major development in HCC therapy has been done since 2019. Because of these limitations, our results should be interpreted with caution. A future challenge will be done to validate, through larger prospective studies, whether the administration of albumin preparations or branched-chain amino acid supplements in patients with liver cirrhosis can reduce the incidence of HCC, and whether such interventions can improve prognosis in patients already diagnosed with HCC.

## 5. Conclusions

Low albumin levels may not only indicate deteriorating liver function but also increase the risk of cancer invasion and metastasis. In HCC patients with Alb < 3.5 g/dL, nutritional improvement and albumin supplementation may reduce intrahepatic metastasis and invasion, ultimately improving prognosis.

## Figures and Tables

**Table 1:** Clinical background of 78 patients in Groups A and T.

Characteristic	A Group (n = 42)	T Group (n = 36)	*p*-Value *
Age, years	70.5 ± 8.5	75.6 ± 7.6	0.006
Sex (M/F)	30/12	25/11	0.38
Etiology of HCC			
HCV/HBV/Alcohol/NASH/AIH /Unknown	15/6/12/4/1/4	16/5/7/0/1/7	0.15
^†^ PS (0/1/2/3/4)	31/7/3/1/0	12/7/5/12/0	<0.001
^††^ HCC Stage (I/II/III/IV)	11/18/10/3	3/10/15/8	<0.001
Maximum tumor diameter (mm)	34.8 ± 28.5	56.6 ± 36.7	0.005
^†††^ Treatment modality			
Ope/RFA/TACE/Chemo/None	19/11/12/0/0	9/5/19/1/2	0.003
Total Bilirubin (mg/dL)	1.3 ± 0.3	0.6 ± 0.2	0.0001
Albumin (g/dL)	3.8 ± 0.2	3.1 ± 0.3	0.0001
Platelet count (×10^4^/μL)	17.2 ± 28.2	20.7 ± 11.1	0.46
Prothrombin time (%)	75.2 ± 12.2	79.2 ± 15.0	0.18

Values are expressed as means ± standard deviation, unless otherwise indicated. ^†^ PS: Performance status was evaluated according to the Eastern Cooperative Oncology Group (ECOG) criteria. ^††^ HCC stage: HCC stage was assessed based on the TNM staging system (8th edition of the UICC/AJCC). ^†††^ Treatment modality: Ope = surgical resection; RFA = radiofrequency ablation; TACE = transarterial chemoembolization; Chemo = systemic chemotherapy; None = no treatment. * χ^2^ test.

**Table 2:** Clinical background of 36 patients after propensity score matching.

Characteristic	A Group (n = 18)	T Group (n = 18)	*p*-Value *
Age, years	75.0 ± 6.0	72.5 ± 6.9	0.77
Sex (M/F)	7/11	4/14	0.28
Etiology of HCC			
HCV/HBV/Alcohol/NASH/AIH /Unknown	9/1/1/2/1/3	7/2/4/0/1/4	0.74
^†^ Performance Status (0/1/2/3/4)	11/4/2/1/0	12/3/2/1/0	1.0
^††^ HCC Stage (I/II/III/IV)	2/9/6/1	2/8/6/2	0.41
Maximum tumor diameter (mm)	27.0 ± 34.5	50.0 ± 38.9	0.20
Treatment modality			
^†††^ Ope/RFA/TACE/Chemo/None	9/3/6/0/0	7/4/6/1/0	0.73
Total Bilirubin (mg/dL)	1.2 ± 0.22	0.6 ± 0.20	<0.001
Albumin (g/dL)	3.8 ± 0.15	3.2 ± 0.27	<0.001
Platelet count (×10^4^/μL)	11.6 ± 41.7	19.9 ± 12.2	0.87
Prothrombin time (%)	74.3 ± 10.9	84.3 ± 11.7	0.03

Values are expressed as means ± standard deviation, unless otherwise indicated. ^†^ PS: Performance status was evaluated according to the Eastern Cooperative Oncology Group (ECOG) criteria. ^††^ HCC stage: HCC stage was assessed based on the TNM staging system (8th edition of the UICC/AJCC). ^†††^ Treatment modality: Ope = surgical resection; RFA = radiofrequency ablation; TACE = transarterial chemoembolization; Chemo = systemic chemotherapy; None = no treatment. * χ^2^ test.

**Table 3:** Clinical background of 44 patients receiving curative treatment.

Characteristic	A Group (n = 30)	T Group (n = 14)	*p*-Value *
Age, years	70 ± 7.9	73 ± 5.7	0.2
Sex (M/F)	20/10	11/3	0.46
Etiology of HCC			
HCV/HBV/Alcohol/NASH/AIH /Unknown	9/5/9/3/1/3	6/2/3/0/1/2	0.52
^†^ Performance Status (0/1/2/3/4)	24/3/3/0/0	9/1/1/3/0	0.11
^††^ HCC Stage (I/II/III/IV)	10/15/5/0	3/6/5/0	0.16
Maximum tumor diameter (mm)	24.3 ± 10.6	37.1 ± 22.3	0.06
^†††^ Treatment modality			
Ope/RFA	19/11	9/5	0.95
Total Bilirubin (mg/dL)	1.2 ± 0.2	0.6 ± 0.2	0.0001
Albumin (g/dL)	3.8 ± 0.2	3.1 ± 0.2	0.0001
Platelet count (×10^4^/μL)	13.1 ± 6.7	19.3 ± 12.4	0.10
Prothrombin time (%)	75.4 ± 12.5	76.5 ± 13.0	0.80

Values are expressed as means ± standard deviation, unless otherwise indicated. ^†^ PS: Performance status was evaluated according to the Eastern Cooperative Oncology Group (ECOG) criteria. ^††^ HCC stage: HCC stage was assessed based on the TNM staging system (8th edition of the UICC/AJCC). ^†††^ Treatment modality: Ope = surgical resection; RFA = radiofrequency ablation; * χ^2^ test.

**Table 4:** Clinical background of 16 patients who underwent RFA.

Characteristic	A Group (n = 11)	T Group (n = 5)	*p*-Value *
Age, years	69 ± 6.4	75 ± 6.4	0.28
Sex (M/F)	8/3	0/5	0.007
Etiology of HCC			
HCV/HBV/Alcohol/NASH/AIH /Unknown	2/3/4/2/0/0	2/1/1/0/0/1	0.59
^†^ Performance Status (0/1/2/3/4)	7/2/2/0/0	2/1/1/1/0	0.35
^††^ HCC Stage (I/II/III/IV)	7/4/0/0	1/3/1/0	0.13
Maximum tumor diameter (mm)	15 ± 6.3	23 ± 5.9	0.44
Total Bilirubin (mg/dL)	1.1 ± 0.14	0.6 ± 0.19	0.0001
Albumin (g/dL)	3.8 ± 0.12	2.9 ± 0.2	0.0001
Platelet count (×10^4^/μL)	13.1 ± 6.7	19.3 ± 12.4	0.74
Prothrombin time (%)	74.3 ± 13.6	78.3 ± 12.6	0.38

Values are expressed as means ± standard deviation, unless otherwise indicated. ^†^ PS: Performance status was evaluated according to the Eastern Cooperative Oncology Group (ECOG) criteria.: ^††^ HCC stage: HCC stage was assessed based on the TNM staging system (8th edition of the UICC/AJCC). * χ^2^ test.

**Table 5:** Clinical background of 28 patients who underwent surgical resection.

Characteristic	A Group (n = 19)	T Group (n = 9)	*p*-Value *
Age, years	71.0 ± 7.2	72.0 ± 5.3	0.49
Sex (M/F)	12/7	6/3	0.86
^†^ Etiology of HCC			
HCV/HBV/Alcohol/NASH/AIH /Unknown	6/3/5/1/1/3	4/1/2/1/0/1	0.69
^††^ Performance Status (0/1/2/3/4)	17/1/1/0/0	7/0/0/2/0	0.18
^†††^ HCC Stage (I/II/III/IV)	3/10/6/0	2/5/2/0	0.61
Maximum tumor diameter (mm)	28 ± 10.6	45 ± 23.3	0.07
Total Bilirubin(mg/dL)	1.3 ± 0.22	0.7 ± 0.15	<0.001
Albumin (g/dL)	3.9 ± 0.17	3.2 ± 0.23	<0.001
Platelet count (×10^4^/μL)	73.3 ± 11.8	11.6 ± 6.4	0.56
Prothrombin time (%)	76.2 ± 12.8	16.4 ± 12.3	0.15

Values are expressed as means ± standard deviation, unless otherwise indicated. ^†^ Etiology of HCC: HCV = hepatitis C virus; HBV = hepatitis B virus; NASH = nonalcoholic steatohepatitis; AIH = autoimmune hepatitis; Unknown = unknown etiology. ^††^ PS: Performance status was evaluated according to the Eastern Cooperative Oncology Group (ECOG) criteria. ^†††^ HCC stage: HCC stage was assessed based on the TNM staging system (8th edition of the UICC/AJCC). * χ2 test.

## Data Availability

The original contributions presented in this study are included in the article. Further inquiries can be directed to the corresponding author.

## Abbreviations

The following abbreviations are used in this manuscript:

HCC	Hepatocellular carcinoma
Alb	Albumin
Bil	Bilirubin
OS	Overall survival
PFS	Progression-free survival
ALBI	albumin-bilirubin
PS	Performance status
CT	computed tomography
MRI	magnetic resonance imaging
TMN	the Tumor-Node-Metastasis
UICC	the Union for International Cancer Control
BCLC	the Barcelona Clinic Liver Cancer
AASLD	the American Association for the Study of Liver Diseases
JSH	the Japan Society of Hepatology
Ope	surgical resection
RFA	radiofrequency ablation
TACE	transarterial chemoembolization
PSM	propensity score matching
BCAA	branched-chain amino acids
uPA	urokinase-type plasminogen activator
MMPs	matrix metalloproteinases
EMT	epithelial–mesenchymal transition
